# Sphingomyelin phosphodiesterase 3 methylation and silencing in oral squamous cell carcinoma results in increased migration and invasion and altered stress response

**DOI:** 10.18632/oncotarget.27458

**Published:** 2020-02-04

**Authors:** James Jabalee, Rebecca Towle, James Lawson, Christopher Dickman, Cathie Garnis

**Affiliations:** ^1^Department of Integrative Oncology, British Columbia Cancer Research Centre, Vancouver, BC, Canada; ^2^Division of Otolaryngology, Department of Surgery, University of British Columbia, Vancouver, BC, Canada

**Keywords:** oral squamous cell carcinoma, DNA methylation, *SMPD3*, erlotinib, cancer progression

## Abstract

Neutral sphingomyelinase 2 (nSMase2), the product of the sphingomyelin phosphodiesterase 3 (*SMPD3*) gene, catalyzes the hydrolysis of sphingomyelin to ceramide. Ceramide acts on various signaling pathways to influence cell proliferation, survival, and stress response. Altered levels of sphingolipids and ceramides have been reported in various cancer types, including oral squamous cell carcinoma (OSCC). OSCC patients exhibit a poor 5-year survival rate of 50%, a figure that has remained stagnant for decades. To overcome this requires a better understanding of the molecular events driving this disease. The molecular analysis of the oral cavity reported here has identified the *SMPD3* promoter region as a site of frequent hypermethylation and downregulation in pre-malignant and malignant tissues as compared with healthy control tissues. While lentivirus-induced overexpression of *SMPD3* in cell models of oral dysplasia and OSCC did not significantly alter proliferation, it did decrease migration and invasion and increased resistance to the epidermal growth factor receptor (EGFR) inhibitor erlotinib. These results suggest that *SMPD3* downregulation is a common event in OSCC progression and may promote the spread of tumor cells.

## INTRODUCTION

Oral squamous cell carcinoma (OSCC) is among the most commonly diagnosed cancer types in the world, with over 300,000 new cases and 145,000 deaths attributed to the disease worldwide each year [1]. OSCC displays a dismal five-year survival rate of approximately 50%, a rate that has not improved in decades [2]. This is due primarily to frequent late-stage diagnoses and high rates of recurrence [3, 4]. To better treat and diagnose OSCC, an improved understanding of the molecular mechanisms underlying tumorigenesis is required. Such knowledge is instrumental to the development of clinical biomarkers, novel therapeutics, and personalized treatments.

We previously performed genome-wide DNA methylation and expression profiling of patient-matched normal, dysplasia, and carcinoma *in situ* (CIS) or OSCC samples obtained from the oral cavity (GEO Accession # GSE46802), with the sphingomyelin phosphodiesterase 3 (*SMPD3)* gene promoter identified as a site of frequent hypermethylation and silencing in dysplasia and CIS/OSCC samples as compared with normal controls [5].

*SMPD3* encodes the protein neutral sphingomyelinase 2 (nSMase2), which hydrolyzes the membrane lipid sphingomyelin to ceramide. Ceramide plays a complex role in the cell by altering the composition of lipid rafts and impinging on various signalling pathways [6, 7]. Initial studies on the role of ceramide found that it induced apoptosis and inhibited the cell cycle in leukemic cells, suggesting a tumor suppressive role [8, 9]. However, subsequent studies have uncovered additional complexity. For instance, ceramide accumulation following nSMase2 activation that was triggered by oxidative stress resulted in apoptosis of human airway epithelial cells, whereas activation of the same enzyme protected neuroblastoma cells from nutrient deprivation-induced cell death [10–12]. nSMase2-mediated generation of ceramide may be further influenced by expression of other ceramide-metabolizing enzymes, such as ceramidases, ceramide kinase and glucosyltransferase, and enzymes involved in *de novo* ceramide synthesis [13]. Recent work has shown a role for nSMase2 in the production of extracellular vesicles. These small vesicles are loaded with specific cargo molecules, including proteins and microRNAs, before being extruded into the extracellular space where they are taken up by neighboring cells. Extracellular vesicles play a key role in regulating the tumor microenvironment and thereby influence tumor progression in a manner dependent upon their cargo [14]. Thus, the role of nSMase2 is context-dependent and has not been examined in OSCC cells.

Herein, we show that *SMPD3* is commonly hypermethylated and its expression is downregulated during oral tumorigenesis. Overexpression of *SMPD3* decreases the migration and invasion of OSCC cell lines and alters their response to stress in a context-dependent manner.

## RESULTS

### SMPD3 promoter CpG island hypermethylation and gene silencing is common in patients with oral dysplasia and CIS/OSCC

**Figure 1:**
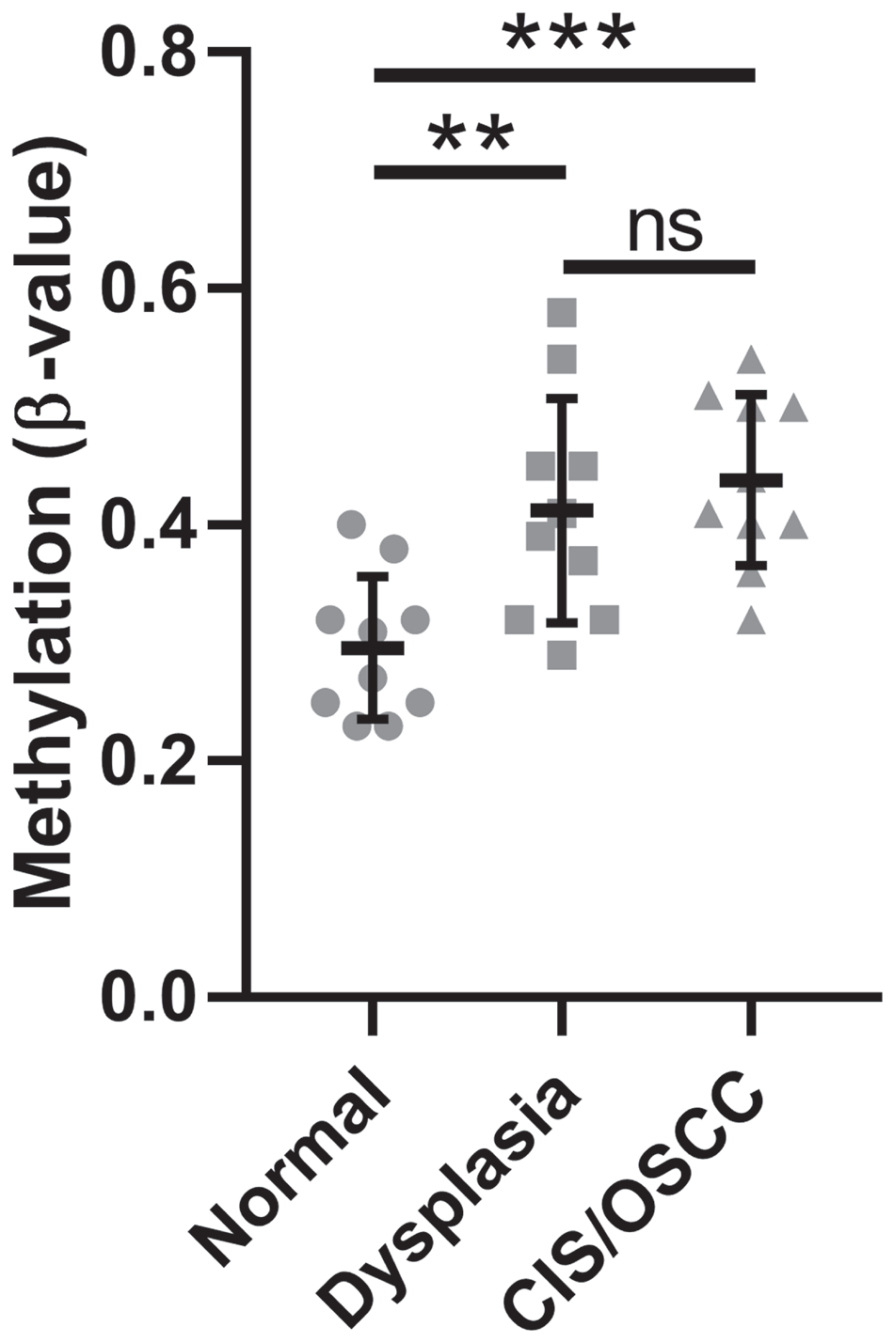
Methylation of the *SMPD3* promoter CpG island is increased in oral dysplasia and cancer tissues compared with normal tissues. The mean β-value for CpGs within the *SMPD3* promoter CpG island across patient-matched samples was measured for normal, dysplastic, and carcinoma *in situ*/oral squamous cell carcinoma tissues from each of 10 patients. Bars represent the mean and standard deviation. Significance was determined using a one-way ANOVA with post-hoc Tukey’s multiple comparisons test. ^**^ *P* < 0.01. ^***^ *P* < 0.001. Abbreviations: ns, not significant; CIS/OSCC, carcinoma *in situ*/oral squamous cell carcinoma.

We previously performed whole-genome methylation and gene expression profiling of patient-derived oral normal, dysplasia, and CIS/OSCC samples [5]. Of the seven CpG sites in the data set, we included only those five known to be located within the promoter CpG island of *SMPD3* in our analysis (cg00891541, cg15201635, cg10556064, cg22116290, cg23758485) as hypermethylation of this genomic region has been linked to cancer progression through repression of tumor suppressor genes [15]. The average β-value of these CpGs across all normal, dysplasia, and CIS/OSCC samples were 0.30 ± 0.06, 0.41 ± 0.09, and 0.44 ± 0.07, respectively (Figure 1). A statistically significant difference in the distribution of β-values was observed between normal vs. dysplasia (one-way ANOVA with Tukey’s multiple comparisons test; F (2, 27) = 9.564, *p* = 0.007 and q (27) = 4.745, *p* = 0.007) and normal vs. CIS/OSCC (q (27) = 5.809, *p* = 0.001), but not dysplasia vs. tumor (q (27) = 1.064, *p* = 0.735), suggesting that *SMPD3* hypermethylation occurs in the dysplasia stage and persists as cells progress towards cancer. Integration of methylation and paired gene expression data revealed that 4/10 dysplasia and 6/10 CIS/OSCC samples show both promoter hypermethylation (defined as a β-value increase of ≥0.15 in at least one CpG) and ≥2-fold lower expression compared with normal in the same patient sample (Table 1). In contrast, hypomethylation (β-value decrease of ≥0.15 in at least one CpG) was not observed for any sample.

**Table 1:** Expression and methylation of *SMPD3* in patient-matched dysplasia and CIS/OSCC samples

	Dysplasia	CIS/OSCC
Hypermethylated^a^	6	10
Downregulated^b^	5	6
Hypermethylated and downregulated in the same patient	4	6

^a^Δβ-value of ≥0.15 compared with adjacent normal tissue in at least one CpG within the *SMPD3* promoter. ^b^Fold-change of ≥2 compared with adjacent normal tissue in at least one *SMPD3* probe. *N* = 10.

**Figure 2:**
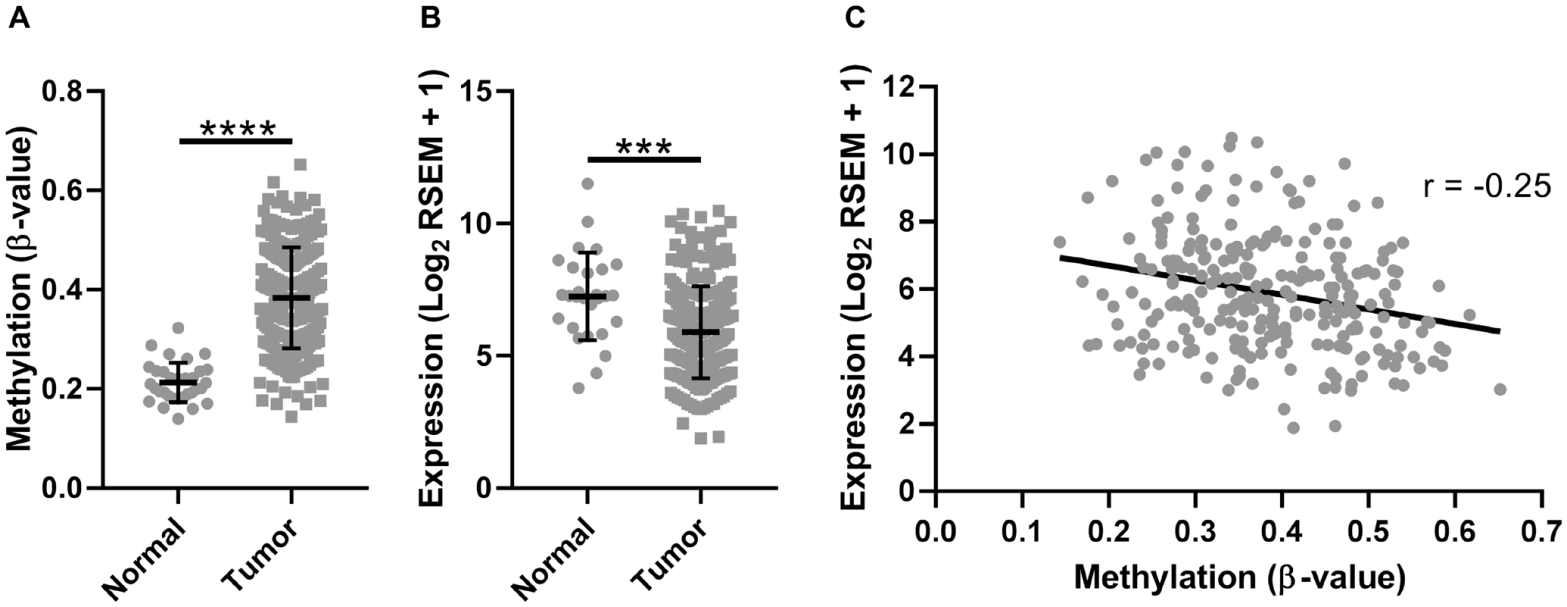
The promoter CpG island of *SMPD3* is hypermethylated and its expression is downregulated in oral cancer samples profiled by The Cancer Genome Atlas. (**A**) The mean β-value for CpGs within the *SMPD3* promoter CpG island across unpaired normal (*N* = 32) and tumor (*N* = 261) samples. (**B**) *SMPD3* expression in unpaired normal (*N* = 28) and tumor (*N* = 252) samples. (**C**) Correlation between gene expression (log_2_ RSEM) and methylation (average β-value within promoter CpG island). *N* = 252. Bars represent the mean and standard deviation (A and B). Statistical significance was determined using a Student’s *t*-test (B) or Student’s *t*-test with Welch’s correction for unequal variances (A). ^***^ *P* < 0.001; ^****^ *P* < 0.0001.

To determine if the observed trend in *SMPD3* promoter methylation and expression was specific to our cohort or was representative of oral cancer more generally, we examined a publicly available TCGA data set of head and neck cancers profiled for both methylation (Illumina 450k array) and gene expression (Illumina HiSeq RNA-seq). We included only those samples corresponding to the oral cavity (buccal mucosa, hard palate, floor of mouth, oral tongue, oral cavity, and alveolar ridge). The Illumina 450k array contains 39 probes corresponding to *SMPD3*, compared with only seven for the 27k array. For consistency, we included only those probes corresponding to CpG island 2 (cpgiid 9439), which includes the five probes used in our initial analysis plus an additional 12 probes (cg04464276, cg08890345, cg0594840, cg05144928, cg12136772, cg05676789, cg16694003, cg03412735, cg08694014, cg09202227, cg07461772, cg10426893). In agreement with our previous observations, we found a significant increase in the average β-value (Student’s *t*-test with Welch’s correction, t (91.64) = 17.94, *p* < 0.0001; Figure 2A) and concomitant decrease in the average expression of *SMPD3* (Student’s *t*-test, t (278) = 3.922, *p* = 0.0001; Figure 2B) in tumor samples as compared with normal samples. We also observed a significant negative correlation between tumor methylation and gene expression (Pearson correlation, F (1,250) = 16.86, *p* < 0.0001, r = –0.25; Figure 2C). Using only paired data, a β-value increase of ≥0.15 was observed in 23/32 (72%) of patients, whereas a β-value decrease of ≥0.15 was not observed for any patient (Supplementary Figure 1). A ≥2-fold decrease in *SMPD3* expression in the tumor was observed in 16/27 (59%) of patients, whereas a ≥2-fold increase was observed in 3/27 (11%). Of 14 patients with paired methylation and expression data, four (29%) exhibited a concomitant increase in methylation and decrease in expression.

**Figure 3:**
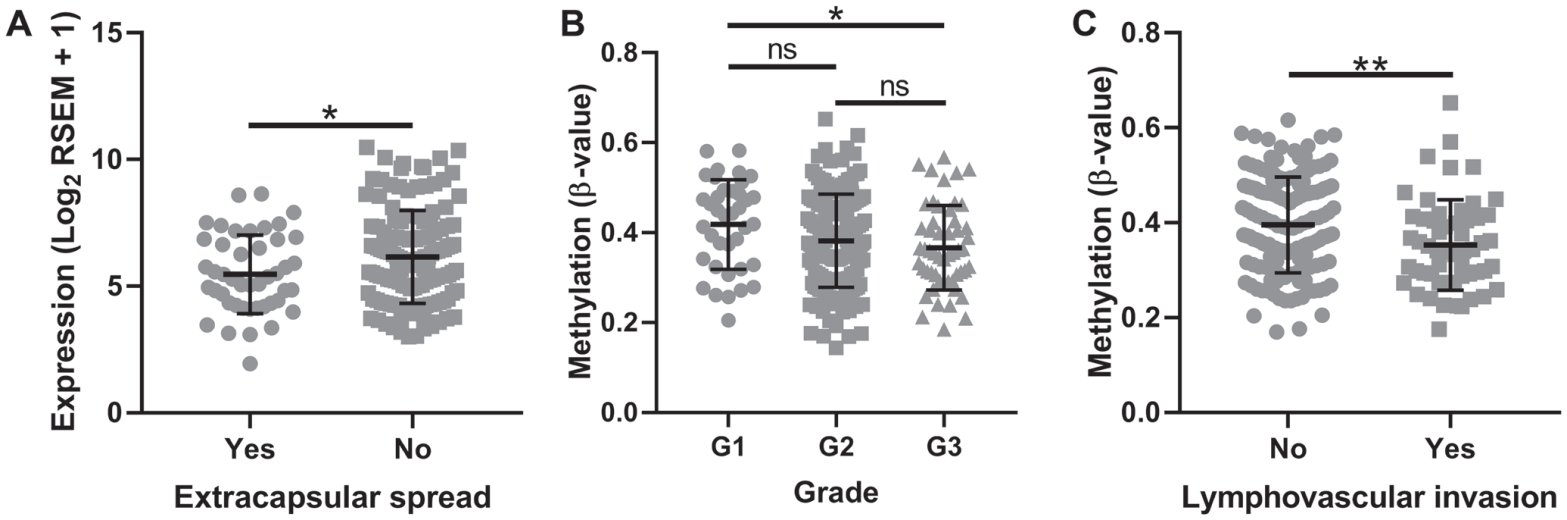
*SMPD3* promoter CpG island methylation and expression correlate with clinical factors as profiled by The Cancer Genome Atlas. (**A**) Tumors displaying extracapsular spread exhibit significantly lower expression of *SMPD3*. (**B**) Methylation of *SMPD3* decreases as tumor grade increases. (**C**) Tumors displaying lymphovascular invasion exhibit significantly lower methylation of *SMPD3*. Bars represent the mean and standard deviation. Statistical significance was determined using Student’s *t*-test (A and C) or one-way ANOVA with post-hoc Tukey’s multiple comparisons test (B). ^*^ *P* < 0.05. ^**^ *P* < 0.01. Abbreviations: ns, not significant.

We further examined TCGA data to determine if tumor *SMPD3* promoter CpG island 2 methylation (using the average β-value of the 17 probes from TCGA as listed above) and *SMPD3* expression correlate with common clinical factors. Interestingly, we found that patients with extracapsular spread exhibited significantly lower *SMPD3* expression in the tumor (Student’s *t*-test, t (177) = 2.254, *p* = 0.025; Figure 3). Further, we found a downward trend in G island 2 methylation as tumor grade increases (one-way ANOVA, F (2,225) = 3.038, *p* = 0.049). Finally, patients with lymphovascular invasion exhibited significantly lower methylation in the tumor (Student’s *t*-test, t (200) = 2.7, *p* = 0.008). Other features, including tumor size, lymph node status, pathological stage, smoking and drinking history, and overall survival, did not correlate significantly with either expression or methylation.

### SMPD3 is methylated and silenced in oral dysplasia and cancer cell lines

**Figure 4:**
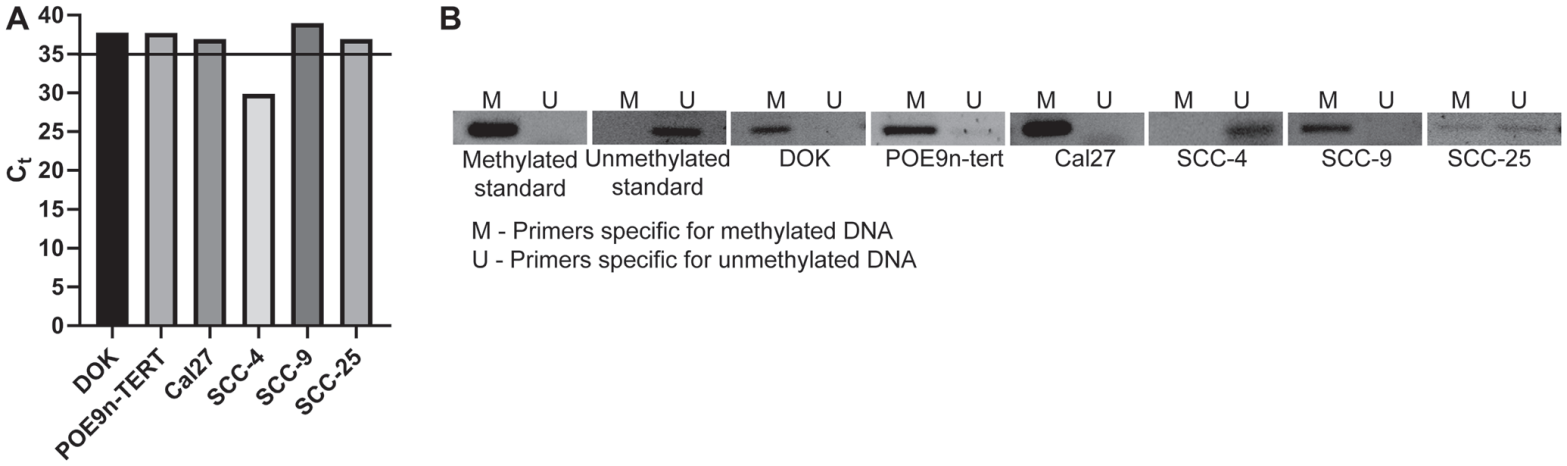
*SMPD3* is methylated and its expression is undetected in a panel of oral dysplasia and cancer cell lines, except for SCC-4. (**A**) *SMPD3* expression was assessed using qRT-PCR. A cycle threshold (Ct) value <35 was considered expressed. (**B**) *SMPD3* methylation was assessed using methylation-specific PCR.

To determine if the observed trends of *SMPD3* hypermethylation and downregulation are true for commonly used OSCC cell lines, we next assessed the methylation and expression status of *SMPD3* in the oral dysplasia lines DOK and POE9n-TERT and the OSCC cell lines Cal27, SCC-4, SCC-9, and SCC-25. qRT-PCR revealed detectable *SMPD3* expression, defined as Ct < 35, in SCC-4 only. Consistent with the expression data, methylation-specific PCR revealed promoter methylation in all lines except SCC-4 (Figure 4). Taken together, our results suggest that promoter hypermethylation and silencing of *SMPD3* are common events in oral tumorigenesis and that they occur early on in cancer progression (i.e., dysplasia).

### SMPD3 decreases migration and invasion of oral dysplasia and OSCC cell lines

**Figure 5:**
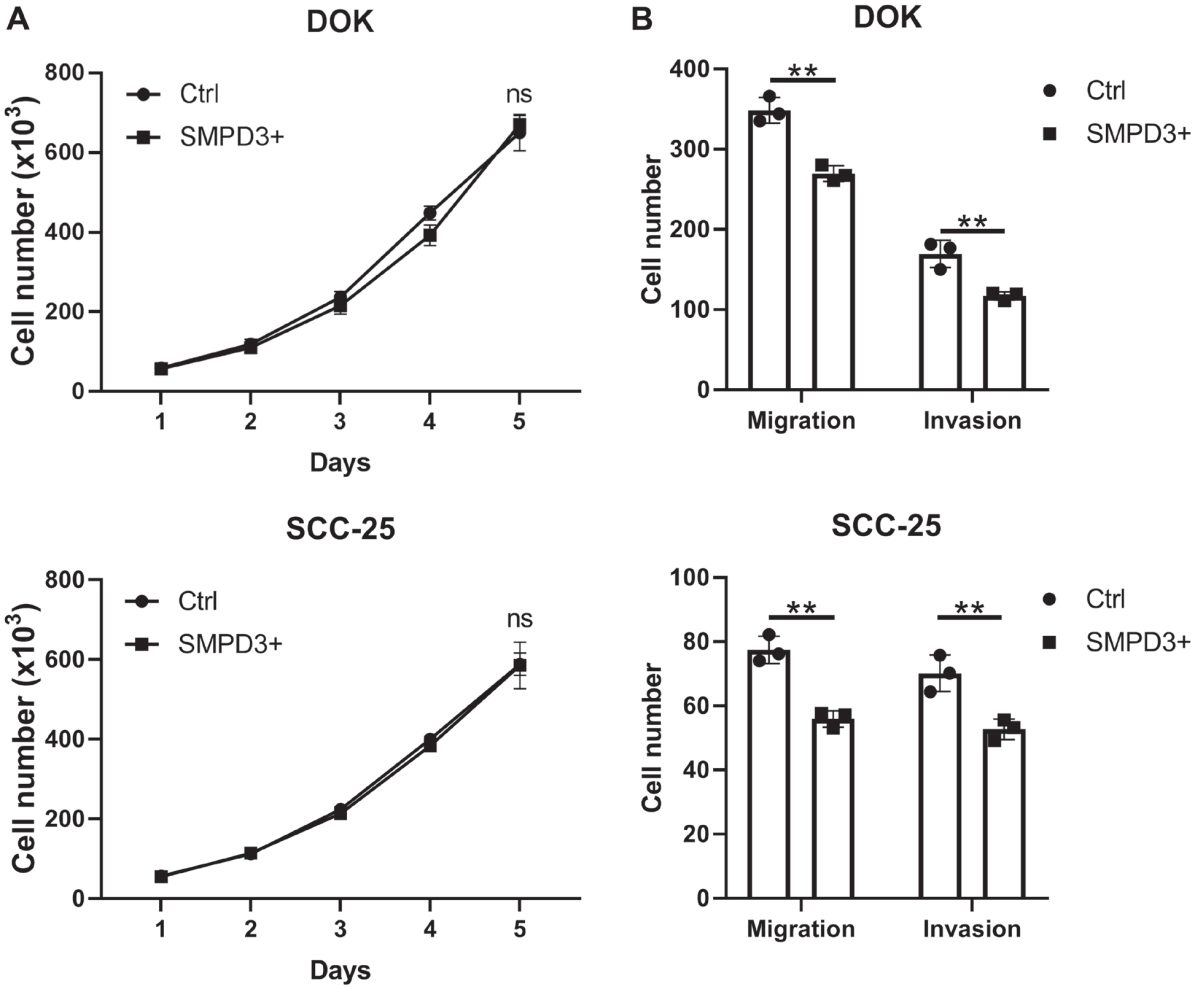
*SMPD3* overexpression has no effect on proliferation but decreases migration and invasion of oral dysplasia and cancer cell lines. (**A**) Cell lines containing a doxycycline-inducible *SMPD3* overexpression vector were treated with zero (Ctrl) or 100 ng/ml (*SMPD3*+) doxycycline, plated in triplicate, and counted every 24 hours using a hemocytometer. (**B**) Ctrl and *SMPD3*+ cells were plated in triplicate in transwell assays with zero or 200 µg/ml Matrigel and grown for 48 hours to assess migration and invasion, respectively. Bars represent the mean and standard deviation. Statistical significance was determined using Student’s *t*-test. ^**^ *P* < 0.01. Abbreviations: ns, not significant.

To further elucidate the role of *SMPD3* in oral dysplasia and cancer, we overexpressed the gene in oral dysplasia (DOK) and carcinoma (SCC-25) cell lines using a doxycycline-inducible vector. To test the effect of *SMPD3* overexpression on proliferation, cells containing the doxycycline-inducible overexpression vector were treated with 0 ng/ml (Ctrl) or 100 ng/ml (*SMPD3*+) doxycycline to activate gene expression. *SMPD3* overexpression did not alter proliferation of DOK or SCC-25 cells over a five-day period (Student’s *t*-test; DOK: t (4) = 0.676, *p* = 0.536; SCC-25: t (4) = 0.071, *p* = 0.947; Figure 5). Migration and invasion were assessed using a transwell invasion assay with zero or 200 µg/ml Matrigel, respectively. *SMPD3* overexpression caused a significant decrease in migration and invasion of DOK (Student’s *t*-test; migration: t (4) = 7.264, *p* = 0.002; invasion: t (4) = 5.132, *p* = 0.007) and SCC-25 (Student’s *t*-test; migration: t (4) = 7.534, *p* = 0.002; invasion: t (4) = 4.616, *p* = 0.010) cells. Doxycycline did not alter the proliferation, migration, or invasion of DOK or SCC-25 cells transfected with control (empty) vector (data not shown).

### SMPD3 does not impact response of oral dysplasia and OSCC cell lines to serum starvation or radiation but increases resistance to erlotinib

**Figure 6:**
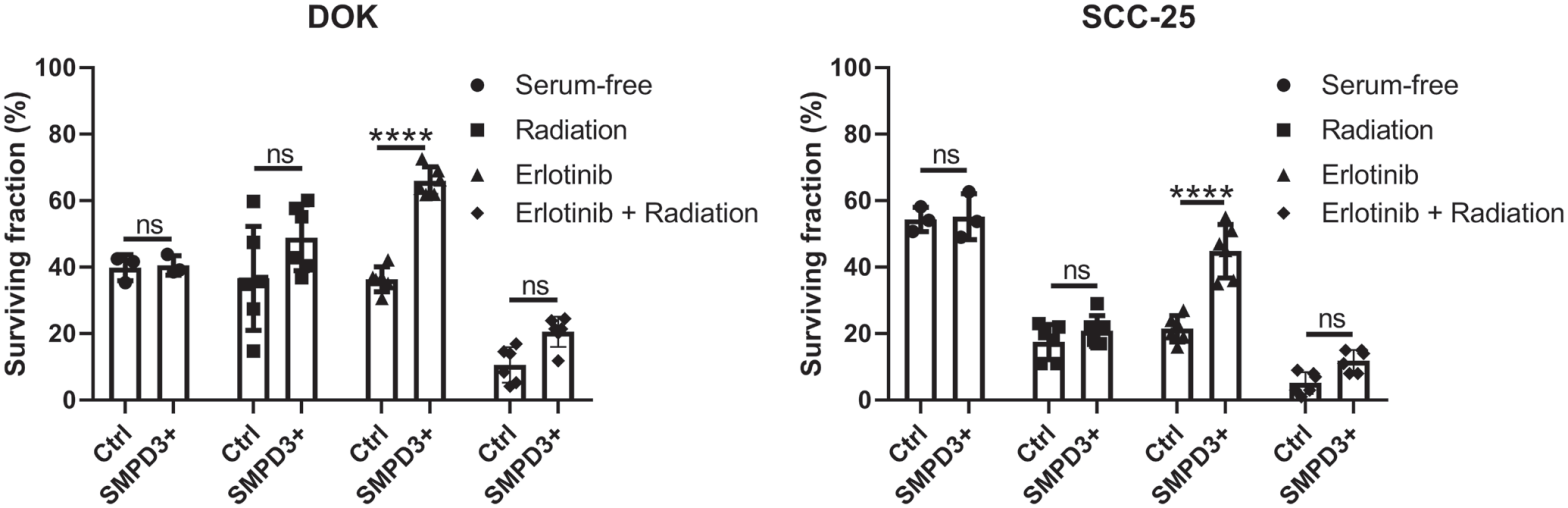
*SMPD3* overexpression did not alter the response of oral dysplasia and cancer cell lines to serum starvation or radiation but improved resistance to the EGFR inhibitor erlotinib. For serum starvation, cells were counted after growing for 48 hours in 10% FBS-containing or serum-free media. The effect of *SMPD3* overexpression on radiation and erlotinib response was assessed using clonogenic assays. Bars represent the mean and standard deviation. Statistical significance was determined using a two-way ANOVA with post-hoc Šidák correction. ^****^ *P* < 0.0001. ns: not significant.

*SMPD3* has been shown to alter the response of cells to various stressors relevant to tumorigenesis. It was recently reported that fibroblasts cultured from *fro/fro* mice (which lack functional *SMPD3*) display increased survival under nutrient deprivation compared with fibroblasts from wild-type mice [16]. Since nutrient stress is common in tumors, we investigated whether *SMPD3* plays a similar role in oral cancer cell lines by comparing the survival of control and *SMPD3*-overexpressing lines after 48 hours in serum-free media. SCC-25 and DOK showed no difference in survival between control and overexpression cells (Student’s *t*-test; DOK: t (4) = 0.250, *p* = 0.815; SCC-25: t (4) = 0.182, *p* = 0.864; Figure 6).

Additional stressors, namely radiation and epidermal growth factor receptor (EGFR) inhibition, were investigated using clonogenic assays. *SMPD3* expression has been shown to alter radiosensitivity in other cancer types and radiation is commonly used to treat OSCC [17]. *EGFR* is commonly amplified in OSCC and has been shown to interact with ceramide in the development of cigarette smoke-induced lung cancer in mice [18]. No survival difference was observed between Ctrl and *SMPD3+* cells for DOK or SCC-25 following irradiation (two-way ANOVA with Šidák’s multiple comparison test; DOK: t (30) = 2.514, *p* = 0.052; SCC-25: t (30) = 1.151, *p* = 0.593; Figure 6). To determine if this is due to upregulation of *SMPD3* in Ctrl cells following irradiation, we irradiated cells with 0, 2, 4, 6, and 8 Gray and assessed *SMPD3* expression after 4 and 24 hours. We did not find evidence that irradiation increases *SMPD3* expression (data not shown). Following treatment with the EGFR inhibitor erlotinib, both DOK and SCC-25 *SMPD3*+ cells displayed improved survival (two-way ANOVA with Šidák’s multiple comparison test; DOK: t (30) = 6.093, *p* < 0.0001; SCC-25: t (30) = 8.060, *p* < 0.0001). When treated with a combination of radiation and erlotinib, *SMPD3*+ cells demonstrated a trend toward increased survival which was not significant (two-way ANOVA with Šidák’s multiple comparison test; DOK: t (30) = 2.056, *p* = 0.139; SCC-25: t (30) = 2.303, *p* = 0.083). Taken together, our results demonstrate that *SMPD3* can protect cells against EGFR inhibition. Given the pro-apoptotic role normally attributed to *SMPD3*, the mechanism by which it protects OSCC cell lines is curious and warrants further investigation.

## DISCUSSION

The standard of care for OSCC patients has not changed for decades and OSCC survival rates remain poor. To remedy this, a deeper understanding of the molecular mechanisms underlying the disease are required. Identification of genes and elucidation of their functions during tumorigenesis are essential steps in the development of novel diagnostics and targeted therapies. We identified the promoter region of *SMPD3* as a site of frequent hypermethylation in patient-derived oral tumors and further analyzed its function in oral dysplasia and tumor cell lines.

Our results suggest that *SMPD3* deregulation is a common event in the formation of oral tumors. Indeed, hypermethylation of *SMPD3* has been reported in various cancer types, including clear cell renal cell carcinoma and hepatocellular carcinoma [19, 20]. In agreement, our analysis of unpaired normal and tumor data from TCGA shows that hypermethylation of *SMPD3* occurs in bladder urothelial carcinoma, breast invasive carcinoma, colon adenocarcinoma, head and neck squamous cell carcinoma, liver hepatocellular carcinoma, kidney renal papillary cell carcinoma, lung adenocarcinoma, lung squamous cell carcinoma, thyroid carcinoma, and uterine endometrial carcinoma, but not prostate cancer (Supplementary Figure 2). A statistically significant decrease in expression was also observed in TCGA data for colon adenocarcinoma, head and neck squamous cell carcinoma, liver hepatocellular carcinoma, lung squamous cell carcinoma, and thyroid carcinoma. Expression was increased in breast and prostate carcinoma, and was unchanged in bladder carcinoma, kidney renal papillary carcinoma, lung adenocarcinoma, and uterine endometrial carcinoma. Although mutations in *SMPD3* affect ~5% of acute myeloid leukemia and acute lymphocytic leukemia patients, mutations were found in only 1/279 (0.4%) tumors in the TCGA head and neck cancer dataset, with copy number variations present in only 3/279 (1%) [21]. Thus, it appears as though hypermethylation is the primary mechanism underlying *SMPD3* deregulation in OSCC and possibly other cancer types.

We further observed a statistically significant correlation between *SMPD3* expression or methylation and various clinical factors. Intriguingly, decreased *SMPD3* expression was found to correlate with the presence of extracapsular spread. Extracapsular spread occurs when cancer cells in a lymph node continue to migrate outside of the capsule of the node, is a late step in the process of distant metastasis, and is associated with poor outcome of head and neck cancer patients [22]. This observation agrees with our *in vitro* studies, which showed that cells that don’t express *SMPD3* exhibit higher levels of migration and invasion compared with *SMPD3* overexpression cells. In addition, we found that *SMPD3* methylation correlates with tumor grade and lymphovascular invasion. Lymphovascular invasion refers to the spread of tumor cells into the blood and lymph, precedes the formation of lymph node metasteses, and is an early step in the process of distant metastasis. However, the direction of this correlation was unexpected, as methylation decreased with increasing grade and was low in tumors with lymphovascular invasion. Although a biological explanation for these observations eludes us, technical factors may play a role. For instance, tumor grade and lymphovascular invasion (and extracapsular spread, as discussed above) are subject to a moderate to large degree of interobserver variability [23–25]. Sampling error, in which a small section of tumor is not representative of the tumor at large, may also play a role; in breast cancer, sampling error has been suggested to account for the observation that not all tumors with lymph node metastases exhibit lymphovascular invasion [26]. Additional studies on the correlation between *SMPD3* expression and methylation with clinical factors are therefore required to confirm these results. *SMPD3* expression and methylation did not significantly correlate with patient survival. However, when the dataset was expanded to include all head and neck samples, not only those corresponding to the oral cavity, we found that patients whose tumors exhibited downregulation of *SMPD3* compared with normal tissue exhibited worse overall survival than patients whose tumors had no difference or which exhibited upregulation of *SMPD3* (Gehan-Breslow-Wilcoxon test, χ^2^ (1) = 6.311, *p* = 0.012). Methylation did not significantly correlate with survival of head and neck cancer patients.

*SMPD3* encodes the protein neutral sphingomyelinase 2 (nSMase2) which catalyzes the breakdown of the membrane lipid sphingomyelin to ceramide [27]. Ceramide regulates numerous cell processes, including confluency-induced growth arrest, apoptosis, and the inward budding of the multivesicular body membrane to form extracellular vesicles [7, 28]. Importantly, the roles of ceramide are independent from one another, and the result of ceramide generation is context-dependent. For instance, nSMase2-generated ceramide is greatly increased in MCF7 cells at confluency, resulting in confluency-induced growth arrest but not apoptosis, whereas nSMase2-generated ceramide results in the apoptosis of human airway epithelial cells under H_2_O_2_ or cigarette smoke-induced oxidative stress [10, 11, 29]. In each case, a common event (nSMase2-induced ceramide generation) leads to a different outcome.

We likewise found the effect of *SMPD3* overexpression on proliferation and stress response to be context-dependent, depending on the cell line under study and the stressor itself. While previous studies found that *SMPD3* overexpression was sufficient to slow the proliferation rate of various cancer cell lines, including the breast cancer line MCF-7, the osteosarcoma line F4328, and the hepatocellular carcinoma line JHH-7, we found no effect on proliferation of either oral dysplasia or OSCC cell lines [20, 21, 27]. However, our data suggest that *SMPD3* does inhibit the capacity of cells for migration and invasion, in agreement with previous studies [30].

Contrary to previous reports, which describe a pro-apoptotic role for *SMPD3*, we found that overexpression had no effect on survival under cell stress conditions, with the exception of treatment with the EGFR inhibitor erlotinib, as discussed below [16, 17]. Several explanations exist for why *SMPD3* overexpression had no effect in some cases. First, nSMase2 activity is regulated by various conditions, including the presence of specific stressors, cytokines, post-translational modifications including phosphorylation and palmitoylation, and access to substrate [10, 11, 27, 29, 31–35]. Thus, overexpression of *SMPD3* does not guarantee an increase in nSMase2 activity and ceramide generation. Second, even if nSMase2 activity is significantly increased by *SMPD3* overexpression, the outcome can be modified by the expression of ceramide metabolizing enzymes, which can prevent ceramide accumulation, by the subcellular localization of ceramide production, and by the expression of other effectors, such as pro- and anti-apoptotic proteins [31, 36–38].

We further observed a consistent increase in survival of *SMPD3*+ cells as compared with control cells following treatment with the EGFR inhibitor erlotinib. A similar phenomenon has been described in lung cancer, whereby cells under oxidative stress are resistant to tyrosine kinase inhibitors, including erlotinib, due to abnormal EGFR activation and trafficking that results from its localization to ceramide-rich rafts [39]. Erlotinib has been shown to increase oxidative stress in OSCC cell lines, suggesting a similar mechanism may be at play in the cell lines used here [40]. Additional study is required to determine whether *SMPD3* overexpression helps to sustain EGFR signaling following treatment with erlotinib, whether this sustained signaling is responsible for reduced erlotinib sensitivity in *SMPD3+* cell lines, and whether the limited efficacy of erlotinib in clinical trials of head and neck squamous cell carcinoma may be mediated in part by nSMase2 activation [41–43].

In summary, we have identified the promoter region of *SMPD3* as a common site of hypermethylation and downregulation in oral tumors and in oral dysplasia and cancer cell lines. Our experiments suggest that *SMPD3* regulates the migratory capacity of tumors cells and can alter their response to EGFR-targeted therapy.

## MATERIALS AND METHODS

### DNA methylation and gene expression profiling

Paired normal, dysplasia, and either CIS or OSCC tissues from ten patients were previously profiled for DNA methylation and gene expression using the HumanMethylation27K microarray (Illumina) and 4 × 44K Human Gene Expression microarray (Agilent) [5]. Ethical approval of this study was granted by the Research Ethical Board at the University of British Columbia (ID#: H10-01694) and informed consent was obtained prior to sample collection for each case. CpG’s showing a delta-beta-value (Δβ) ≥0.15 were considered differentially methylated. Similarly, genes showing a ≥2-fold change in expression were considered differentially expressed.

### TCGA data analysis

DNA methylation (from the Illumina 450K Infinium chip), gene expression (from Illumina HiSeq RNA sequencing), and clinical data were obtained from The Cancer Genome Atlas (TCGA; www.cancergenome.nih.gov; RRID: SCR_003193). We use the term OSCC to refer to a subset of head and neck squamous cell carcinomas arising from oral tissues (including the buccal mucosa, floor of the mouth, hard palate, oral tongue, and oral cavity). Data analysis was performed using Excel (Microsoft Corporation; RRID: SCR_016137) and GraphPad Prism version 8.0 (GraphPad Software; RRID: SCR_002798). CpG island methylation was compared between normal and tumor samples, and statistical significance was assessed using a Student’s *t*-test. Correlation of *SMPD3* expression and methylation with clinical variables was performed using a Student’s *t*-test or one-way ANOVA. Welch’s correction was applied when the variables under study exhibited unequal variances as assessed with an *F*-test. Survival was analyzed using a Gehan-Breslow-Wilcoxon test. A *p*-value of <0.05 was considered significant.

### Cell culture

The dysplastic oral cell lines, DOK and POE9n-TERT, were obtained from Sigma and the Harvard Skin Disease Research Center, respectively. DOK cells were cultured in DMEM media supplemented with 10% fetal bovine serum (FBS) and 5 μg/ml hydrocortisone. POE9n-TERT cell were cultured in K-SFM media supplemented with 25 µg/ml bovine pituitary extract and 0.2 ng/ml epidermal growth factor. Four oral cancer cell lines, Cal27 (ATCC^®^ CRL2095™), SCC-4 (ATCC^®^ CRL1624™), SCC-9 (ATCC^®^ CRL1629™) and SCC-25 (ATCC^®^ CRL1628™), were obtained from the American Type Culture Collection and were authenticated by short-tandem repeat profiling prior to receipt. Cal27 were cultured in DMEM media supplemented with 10% FBS. SCC-4, SCC-9, and SCC-25 cells were cultured in DMEM/F12 media supplemented with 10% FBS and 400 ng/ml hydrocortisone. Lentivirus was produced using HEK 293T cells, a generous gift from Dr. Aly Karsan. 293T cells were cultured in DMEM media supplemented with 10% FBS. Cell lines were used for no more than two months after thawing to prevent genetic drift.

### Lentiviral transduction

Doxycycline-inducible overexpression of *SMPD3* was performed using the pINDUCER20 vector (Addgene plasmid #44012; http://n2t.net/addgene:44012) as per the manufacturer’s instructions [44]. Viral production was performed using 293T cells according to the pINDUCER20 method. Cells were selected using Geneticin (400 µg/ml) for one week and *SMPD3* overexpression was induced using 100 ng/ml doxycycline for 24 hours prior to the start of each experiment. Media was refreshed every 48 hours to maintain expression.

### Bisulfite conversion and methylation-specific PCR

DNA was collected in Digestion Buffer (10 mM Tris, 100 mM EDTA, 0.5% SDS, 50 mM NaCl; pH 8.0) containing 100 μg/ml Proteinase K. DNA was incubated at 55°C for 48 hours prior to phenol/chloroform extraction. Bisulfite conversion was performed using the EZ DNA Methylation Kit (Zymo Research) per the manufacturer’s instructions. Three hundred nanograms of genomic DNA were used as input. Methylation-specific primers were designed using MethPrimer software [45]. Sequences are as follows. Methylated forward primer: TCGTTAGTTATTTTCGGTTGGTC; Methylated reverse primer: ACTTATAATTCTTTAAAAACGCGAA; Unmethylated forward primer: TTGTTAGTTATTTTTGGTTGGTTGT; Unmethylated reverse primer: ACTTATAATTCTTTAAAAACACAAA. PCR conditions were as follows: denaturation at 94°C for 2 min followed by 35 cycles of 94°C for 30 sec, 52°C for 30 sec, and 68°C for 45 sec with a final extension step at 68°C for 10 min. PCR fragments were run on a 1.5% agarose gel and visualized using SYBR Safe DNA gel stain.

### Quantitative real-time PCR

To assess *SMPD3* expression in cell lines, RNA was extracted in TRIzol per the manufacturer’s instructions. Complementary DNA was generated from total RNA (600 ng) using the High Capacity Reverse Transcription kit (Applied Biosystems Life Technologies) per the manufacturer’s instructions. Quantitative real-time PCR (qRT-PCR) was performed on a ViiA 7 Real-Time PCR System using TaqMan probes and TaqMan Universal Master Mix II with no UNG (Applied Biosystems). 18S rRNA was used as an endogenous control. qRT-PCR was performed under the following conditions: 50°C for 2 min followed by denaturation at 95°C and 40 cycles of 95°C for 15 sec followed by 60°C for 1 min. Target expression fold change was determined using the double delta cycle threshold (ΔΔCt) method.

### Cell growth and viability assays

Cell growth was determined by plating each treatment in triplicate in a 6-well plate at a density of 25,000 – 50,000 cells/well. Cells were counted daily in triplicate using a standard hemocytometer (Trypan blue exclusion). Data was plotted as the average number of cells/well at each time point. Statistical significance was determined using data from the final day of testing (Student’s *t*-test; *p* < 0.05 considered significant).

Transwell invasion assays were performed by plating 50,000 cells/well in serum-free media with or without addition of 200 µg/ml Matrigel (to examine invasion or migration, respectively). Cells could migrate toward 10% FBS-containing medium for 48 hours before fixation in 0.05% Crystal Violet in 20% ethanol. Non-migrated cells were removed with a cotton swab and each membrane was counted manually as the average of 5 regions (top, bottom, left, right, center).

To assess cell response to serum starvation, cells were seeded at 150,000 cells/well in 6-well plates. Twenty-four hours later, medium was replaced with serum-free medium. Cells were grown for 48 hours and counted in triplicate using a hemocytometer.

Clonogenic assays were used to assess cell response to epidermal growth factor receptor (EGFR) inhibition and radiation. Cells were treated with or without doxycycline for 24 hours, followed by treatment with vehicle (dimethyl sulfoxide) or the EGFR inhibitor erlotinib (5 µM) for 24 hours before being plated into 35 × 10 mm plates at a density of 1000 cells/well. Cells were allowed to adhere to the plates and were then treated with 0 or 2 Gray radiation. Cells were grown for 8–10 days after which colonies were stained with 0.05% crystal violet in 20% ethanol and colonies with ≥50 cells were counted. The surviving fraction was calculated relative to untreated controls (0 Gy, 0 µM erlotinib). Statistical significance of the surviving fraction between control and *SMPD3*+ cells was calculated using two-way ANOVA (*p* < 0.05 considered significant).

## SUPPLEMENTARY MATERIALS



## Abbreviations

ΔΔCT: double delta cycle threshold
CIS: carcinoma *in situ*
Ct: cycle threshold
EGFR: epidermal growth factor receptor
FBS: fetal bovine serum
nSMase2: neutral sphingomyelinase 2
OSCC: oral squamous cell carcinoma
qRT-PCR: quantitative real-time PCR
SMPD3: sphingomyelin phosphodiesterase 3
TCGA: The Cancer Genome Atlas

## References

[1] Torre LA , Bray F , Siegel RL , Ferlay J , Lortet-Tieulent J , Jemal A . Global cancer statistics, 2012. CA Cancer J Clin. 2015; 65:87–108. 10.3322/caac.21262. 25651787

[2] Funk GF , Karnell LH , Robinson RA , Zhen WK , Trask DK , Hoffman HT . Presentation, treatment, and outcome of oral cavity cancer: a National Cancer Data Base report. Head Neck. 2002; 24:165–180. 10.1002/hed.10004. 11891947

[3] Güneri P , Epstein JB . Late stage diagnosis of oral cancer: Components and possible solutions. Oral Oncol. 2014; 50:1131–1136. 10.1016/j.oraloncology.2014.09.005. 25255960

[4] Wang B , Zhang S , Yue K , Wang XD . The recurrence and survival of oral squamous cell carcinoma: a report of 275 cases. Chin J Cancer. 2013; 32:614–618. 10.5732/cjc.012.10219. 23601241 PMC3845544

[5] Towle R , Truong D , Hogg K , Robinson WP , Poh CF , Garnis C . Global analysis of DNA methylation changes during progression of oral cancer. Oral Oncol. 2013; 49:1033–1042. 10.1016/j.oraloncology.2013.08.005. 24035722

[6] London E . Ceramide selectively displaces cholesterol from ordered lipid domains (rafts) implications for lipid raft structure and function. J Biol Chem. 2004; 279:9997–10004. 10.1074/jbc.M309992200. 14699154

[7] Morad SA , Cabot MC . Ceramide-orchestrated signalling in cancer cells. Nat Rev Cancer. 2013; 13:51–65. 10.1038/nrc3398. 23235911

[8] Jayadev S , Liu B , Bielawska AE , Lee JY , Nazaire F , Pushkareva MY , Obeid LM , Hannun YA . Role for ceramide in cell cycle arrest. J Biol Chem. 1995; 270:2047–2052. 10.1074/jbc.270.5.2047. 7836432

[9] Obeid LM , Linardic CM , Karolak LA , Hannun YA . Programmed cell death induced by ceramide. Science. 1993; 259:1769–1771. 10.1126/science.8456305. 8456305

[10] Levy M , Castillo SS , Goldkorn T . nSMase2 activation and trafficking are modulated by oxidative stress to induce apoptosis. Biochem Biophys Res Commun. 2006; 344:900–905. 10.1016/j.bbrc.2006.04.013. 16631623 PMC4370275

[11] Levy M , Khan E , Careaga M , Goldkorn T . Neutral sphingomyelinase 2 is activated by cigarette smoke to augment ceramide-induced apoptosis in lung cell death. Am J Physiol Lung Cell Mol Physiol. 2009; 297:L125–L33. 10.1152/ajplung.00031.2009. 19395669 PMC2711801

[12] Back MJ , Ha HC , Fu Z , Choi JM , Piao Y , Won JH , Jang JM , Shin IC , Kim DK . Activation of neutral sphingomyelinase 2 by starvation induces cell-protective autophagy via an increase in Golgi-localized ceramide. Cell Death Dis. 2018; 9:670. 10.1038/s41419-018-0709-4. 29867196 PMC5986760

[13] Hannun YA , Obeid LM . The ceramide-centric universe of lipid-mediated cell regulation: stress encounters of the lipid kind. J Biol Chem. 2002; 277:25847–25850. 10.1074/jbc.R200008200. 12011103

[14] Kosaka N , Iguchi H , Hagiwara K , Yoshioka Y , Takeshita F , Ochiya T . Neutral sphingomyelinase 2 (nSMase2)-dependent exosomal transfer of angiogenic microRNAs regulate cancer cell metastasis. J Biol Chem. 2013; 288:10849–10859. 10.1074/jbc.M112.446831. 23439645 PMC3624465

[15] Jones PA , Baylin SB . The epigenomics of cancer. Cell. 2007; 128:683–692. 10.1016/j.cell.2007.01.029. 17320506 PMC3894624

[16] Garoby-Salom S , Rouahi M , Mucher E , Auge N , Salvayre R , Negre-Salvayre A . Hyaluronan synthase-2 upregulation protects smpd3-deficient fibroblasts against cell death induced by nutrient deprivation, but not against apoptosis evoked by oxidized LDL. Redox Biol. 2015; 4:118–126. 10.1016/j.redox.2014.12.004. 25555205 PMC4309855

[17] Haimovitz-Friedman A , Kan CC , Ehleiter D , Persaud RS , Mcloughlin M , Fuks Z , Kolesnick RN . Ionizing radiation acts on cellular membranes to generate ceramide and initiate apoptosis. J Exp Med. 1994; 180:525–535. 10.1084/jem.180.2.525. 8046331 PMC2191598

[18] Goldkorn T , Filosto S , Chung S . Lung injury and lung cancer caused by cigarette smoke-induced oxidative stress: molecular mechanisms and therapeutic opportunities involving the ceramide-generating machinery and epidermal growth factor receptor. Antioxid Redox Signal. 2014; 21:2149–74. 10.1089/ars.2013.5469. 24684526 PMC4215561

[19] Wang J , Li J , Gu J , Yu J , Guo S , Zhu Y , Ye D . Abnormal methylation status of FBXW10 and SMPD3, and associations with clinical characteristics in clear cell renal cell carcinoma. Oncol Lett. 2015; 10:3073–3080. 10.3892/ol.2015.3707. 26722292 PMC4665406

[20] Revill K , Wang T , Lachenmayer A , Kojima K , Harrington A , Li J , Hoshida Y , Llovet JM , Powers S . Genome-wide methylation analysis and epigenetic unmasking identify tumor suppressor genes in hepatocellular carcinoma. Gastroenterology. 2013; 145:1424–35.e25. 10.1053/j.gastro.2013.08.055. 24012984 PMC3892430

[21] Kim WJ , Okimoto RA , Purton LE , Goodwin M , Haserlat SM , Dayyani F , Sweetser DA , McClatchey AI , Bernard OA , Look AT , Bell DW , Scadden DT , Haber DA . Mutations in the neutral sphingomyelinase gene SMPD3 implicate the ceramide pathway in human leukemias. Blood. 2008; 111:4716–4722. 10.1182/blood-2007-10-113068. 18299447 PMC2343601

[22] Myers JN , Greenberg JS , Mo V , Roberts D . Extracapsular spread: a significant predictor of treatment failure in patients with squamous cell carcinoma of the tongue. Cancer. 2001; 92:3030–3036. 10.1002/1097-0142(20011215)92:12<3030::aid-cncr10148>3.0.co;2-p. 11753980

[23] Dik EA , Ipenburg NA , Kessler PA , van Es RJ , Willems SM . The value of histological grading of biopsy and resection specimens in early stage oral squamous cell carcinomas. J Craniomaxillofac Surg. 2018; 46:1001–1006. 10.1016/j.jcms.2018.03.019. 29709328

[24] Harris EI , Lewin DN , Wang HL , Lauwers GY , Srivastava A , Shyr Y , Shakhtour B , Revetta F , Washington MK . Lymphovascular invasion in colorectal cancer: an interobserver variability study. Am J Surg Pathol. 2008; 32:1816–1821. 10.1097/PAS.0b013e3181816083. 18779725 PMC2605104

[25] Lewis JS Jr , Tarabishy Y , Luo J , Mani H , Bishop JA , Leon ME , Prasad ML , Xu H , Di Palma S . Inter-and intra-observer variability in the classification of extracapsular extension in p16 positive oropharyngeal squamous cell carcinoma nodal metastases. Oral Oncol. 2015; 51:985–990. 10.1016/j.oraloncology.2015.08.003. 26293844 PMC4875754

[26] Han JS , Molberg KH , Sarode V . Predictors of invasion and axillary lymph node metastasis in patients with a core biopsy diagnosis of ductal carcinoma *in situ*: an analysis of 255 cases. Breast J. 2011; 17:223–229. 10.1111/j.1524-4741.2011.01069.x. 21545433

[27] Marchesini N , Luberto C , Hannun YA . Biochemical properties of mammalian neutral sphingomyelinase2 and its role in sphingolipid metabolism. J Biol Chem. 2003; 278:13775–13783. 10.1074/jbc.M212262200. 12566438

[28] Trajkovic K , Hsu C , Chiantia S , Rajendran L , Wenzel D , Wieland F , Schwille P , Brügger B , Simons M . Ceramide triggers budding of exosome vesicles into multivesicular endosomes. Science. 2008; 319:1244–1247. 10.1126/science.1153124. 18309083

[29] Marchesini N , Osta W , Bielawski J , Luberto C , Obeid LM , Hannun YA . Role for mammalian neutral sphingomyelinase 2 in confluence-induced growth arrest of MCF7 cells. J Biol Chem. 2004; 279:25101–25111. 10.1074/jbc.M313662200. 15051724

[30] Qin J , Kilkus J , Dawson G . The hyaluronic acid inhibitor 4-methylumbelliferone is an NSMase2 activator—role of Ceramide in MU anti-tumor activity. Biochim Biophys Acta. 2016; 1861:78–90. 10.1016/j.bbalip.2015.11.001. 26548718 PMC4691382

[31] Tani M , Hannun YA . Neutral sphingomyelinase 2 is palmitoylated on multiple cysteine residues. Role of palmitoylation in subcellular localization. J Biol Chem. 2007; 282:10047–10056. 10.1074/jbc.M611249200. 17272284

[32] Filosto S , Fry W , Knowlton AA , Goldkorn T . Neutral sphingomyelinase 2 (nSMase2) is a phosphoprotein regulated by calcineurin (PP2B). J Biol Chem. 2010; 285:10213–10222. 10.1074/jbc.M109.069963. 20106976 PMC2856226

[33] Filosto S , Ashfaq M , Chung S , Fry W , Goldkorn T . Neutral sphingomyelinase 2 activity and protein stability are modulated by phosphorylation of five conserved serines. J Biol Chem. 2012; 287:514–522. 10.1074/jbc.M111.315481. 22074919 PMC3249105

[34] Ravid T , Tsaba A , Gee P , Rasooly R , Medina EA , Goldkorn T . Ceramide accumulation precedes caspase-3 activation during apoptosis of A549 human lung adenocarcinoma cells. Am J Physiol Lung Cell Mol Physiol. 2003; 284:L1082–L92. 10.1152/ajplung.00172.2002. 12576296 PMC4370276

[35] Castillo SS , Levy M , Thaikoottathil JV , Goldkorn T . Reactive nitrogen and oxygen species activate different sphingomyelinases to induce apoptosis in airway epithelial cells. Exp Cell Res. 2007; 313:2680–2686. 10.1016/j.yexcr.2007.04.002. 17498692

[36] Liu YY , Han TY , Giuliano AE , Ichikawa S , Hirabayashi Y , Cabot MC . Glycosylation of ceramide potentiates cellular resistance to tumor necrosis factor-α-induced apoptosis. Exp Cell Res. 1999; 252:464–470. 10.1006/excr.1999.4649. 10527636

[37] Bruce CR , Risis S , Babb JR , Yang C , Kowalski GM , Selathurai A , Lee-Young RS , Weir JM , Yoshioka K , Takuwa Y , Meikle PJ , Pitson SM , Febbraio MA . Overexpression of sphingosine kinase 1 prevents ceramide accumulation and ameliorates muscle insulin resistance in high-fat diet–fed mice. Diabetes. 2012; 61:3148–3155. 10.2337/db12-0029. 22961081 PMC3501880

[38] Zhang J , Alter N , Reed JC , Borner C , Obeid LM , Hannun YA . Bcl-2 interrupts the ceramide-mediated pathway of cell death. Proc Natl Acad Sci U S A. 1996; 93:5325–5328. 10.1073/pnas.93.11.5325. 8643573 PMC39244

[39] Filosto S , Khan EM , Tognon E , Becker C , Ashfaq M , Ravid T , Goldkorn T . EGF receptor exposed to oxidative stress acquires abnormal phosphorylation and aberrant activated conformation that impairs canonical dimerization. PLoS One. 2011; 6:e23240. 10.1371/journal.pone.0023240. 21853092 PMC3154401

[40] Orcutt KP , Parsons AD , Sibenaller ZA , Scarbrough PM , Zhu Y , Sobhakumari A , Wilke WW , Kalen AL , Goswami P , Miller FJ Jr , Spitz DR , Simons AL . Erlotinib-mediated inhibition of EGFR signaling induces metabolic oxidative stress through NOX4. Cancer Res. 2011; 71:3932–3940. 10.1158/0008-5472.CAN-10-3425. 21482679 PMC3217301

[41] Martins RG , Parvathaneni U , Bauman JE , Sharma AK , Raez LE , Papagikos MA , Yunus F , Kurland BF , Eaton KD , Liao JJ , Mendez E , Futran N , Wang DX , et al. Cisplatin and radiotherapy with or without erlotinib in locally advanced squamous cell carcinoma of the head and neck: a randomized phase II trial. J Clin Oncol. 2013; 31:1415–1421. 10.1200/JCO.2012.46.3299. 23460709

[42] Massarelli E , Lin H , Ginsberg LE , Tran HT , Lee JJ , Canales JR , Williams MD , Blumenschein GR Jr , Lu C , Heymach JV , Kies MS , Papadimitrakopoulou V . Phase II trial of everolimus and erlotinib in patients with platinum-resistant recurrent and/or metastatic head and neck squamous cell carcinoma. Ann Oncol. 2015; 26:1476–1480. 10.1093/annonc/mdv194. 26025965 PMC4855241

[43] William WN Jr , Papadimitrakopoulou V , Lee JJ , Mao L , Cohen EE , Lin HY , Gillenwater AM , Martin JW , Lingen MW , Boyle JO , Shin DM , Vigneswaran N , Shinn N , et al. Erlotinib and the risk of oral cancer: the erlotinib prevention of oral cancer (EPOC) randomized clinical trial. JAMA Oncol. 2016; 2:209–216. 10.1001/jamaoncol.2015.4364. 26540028 PMC4771491

[44] Meerbrey KL , Hu G , Kessler JD , Roarty K , Li MZ , Fang JE , Herschkowitz JI , Burrows AE , Ciccia A , Sun T , Schmitt EM , Bernardi RJ , Fu X , et al. The pINDUCER lentiviral toolkit for inducible RNA interference *in vitro* and *in vivo* . Proc Natl Acad Sci U S A. 2011; 108:3665–3670. 10.1073/pnas.1019736108. 21307310 PMC3048138

[45] Li LC , Dahiya R . MethPrimer: designing primers for methylation PCRs. Bioinformatics. 2002; 18:1427–1431. 10.1093/bioinformatics/18.11.1427. 12424112

