# Supplementary material for: Sphingomyelin phosphodiesterase 3 methylation and silencing in oral squamous cell carcinoma results in increased migration and invasion and altered stress response
Source: Oncotarget. 2020 Feb 4;11(5):523–34. doi: 10.18632/oncotarget.27458 (PMC7007297; doi:10.18632/oncotarget.27458)
Supplement: Supplementary file 1 [file oncotarget-11-523-s001.pdf]

Sphingomyelin phosphodiesterase 3 methylation and silencing in oral squamous cell carcinoma results in increased migration and invasion and altered stress response

SUPPLEMENTARY MATERIALS

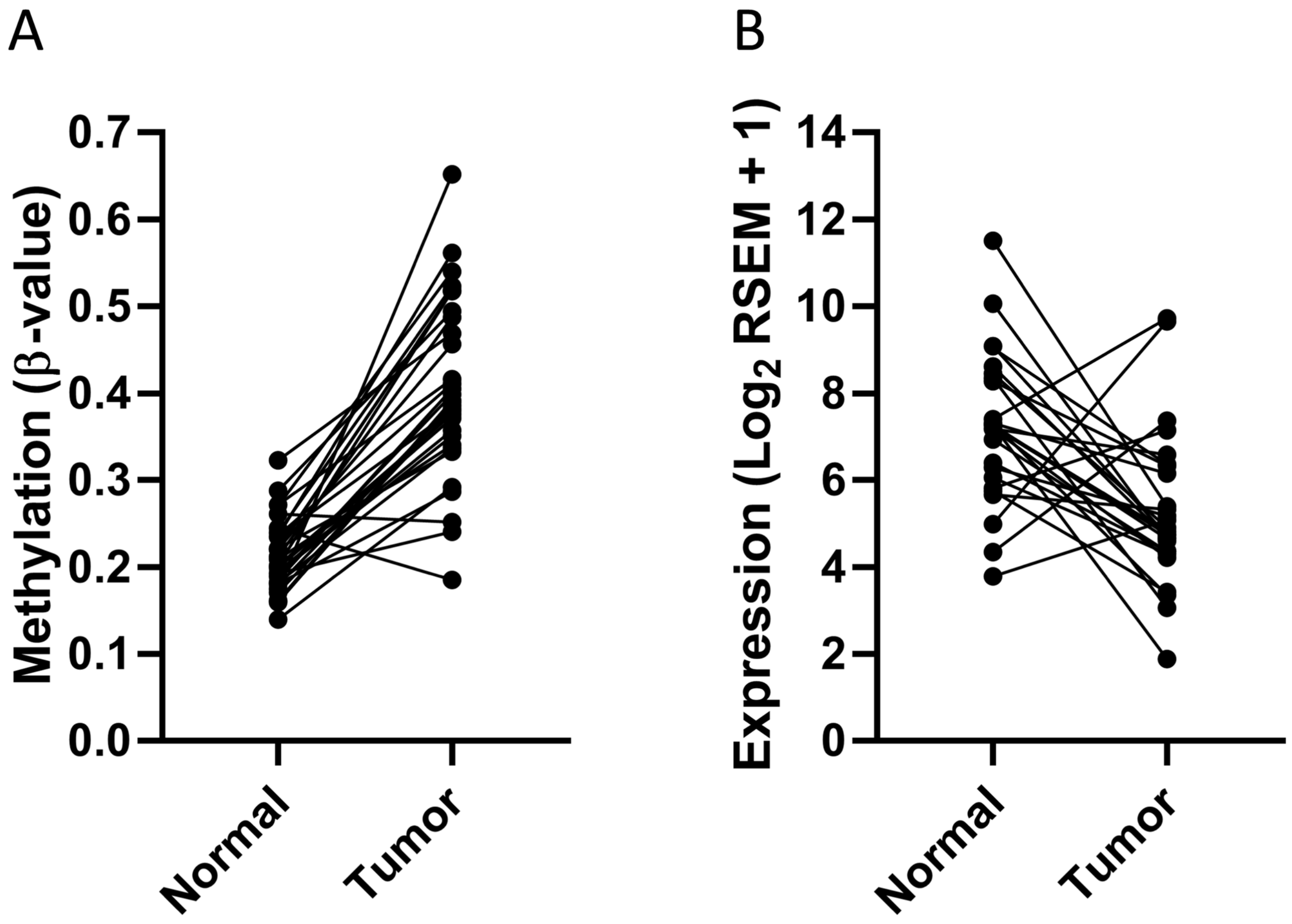

Supplementary Figure 1: The promoter CpG island of *SMPD3* is hypermethylated and its expression is downregulated in paired samples profiled by The Cancer Genome Atlas. (**A**) Average β-value for CpGs within the *SMPD3* promoter CpG island across paired normal and tumor samples ($N = 32$ pairs). (**B**) *SMPD3* expression in paired normal and tumor samples ($N = 14$ pairs).

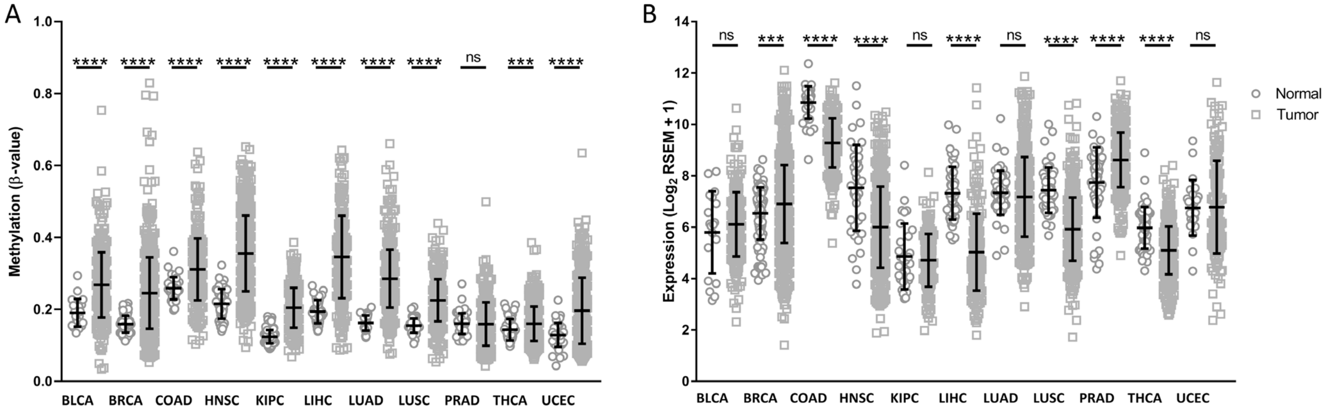

Supplementary Figure 2: *SMPD3* promoter CpG island hypermethylation and downregulation occur in a variety of cancer types as profiled by The Cancer Genome Atlas. (**A**) The average β-value for CpGs within the *SMPD3* promoter CpG island. (**B**) Expression of *SMPD3* in unpaired normal and tumor samples. Bars represent the mean and standard deviation. Statistical significance was determined using a Student's *t*-test (B; BLCA, HNSC, KIPC, THCA) or using a Student's *t*-test with Welch's correction for unequal variance (all other comparisons). $^{***}P < 0.001$. $^{****}P < 0.0001$ ns: not significant. BLCA: Bladder urothelial carcinoma. BRCA: Breast invasive carcinoma. COAD: Colon adenocarcinoma. HNSC: Head and neck squamous cell carcinoma. KIPC: Kidney renal papillary cell carcinoma. LIHC: Liver hepatocellular carcinoma. LUAD: Lung adenocarcinoma. LUSC: Lung squamous cell carcinoma. PRAD: Prostate adenocarcinoma. THCA: Thyroid carcinoma. UCEC: Uterine endometrial carcinoma.
